# Corrective surgery for nipple depression in patients with plasmacytic mastitis – A single-center experience

**DOI:** 10.3389/fmed.2023.1156628

**Published:** 2023-04-06

**Authors:** Yajun Xu, Bu Da, Fengxia Zhao, Mingjuan Gao, Lihua Xue, Hao Zheng, Hongzhi Shi, Lihua Hou, Shan Miao, Xinwei Liu, Yajing Wang, Hong Xu

**Affiliations:** ^1^Affiliated Hospital of Weifang Medical University, School of Clinical Medicine, Weifang Medical University, Weifang, China; ^2^Department of General Surgery, The Third Medical Center, Chinese PLA General Hospital, Beijing, China

**Keywords:** plasma cell mastitis, recurrent, nipple depression correction, surgery, novel surgery

## Abstract

**Background:**

Plasma cell mastitis (PCM) is a complex breast disease in the clinic. Currently, there are no unified diagnostic criteria for the disease and no standard treatment methods. The effects of hormone, Conventional Chinese medicine and other treatments are uncertain, with long treatment duration and notable side effects. Surgery is the preferred treatment, but the recurrence rate after conventional surgery is very high, which may be related to depression of the nipple. This study aimed to evaluate the efficacy of a novel corrective procedure in patients with cellular mastitis and depressed nipples.

**Methods:**

Patients with PCM who received surgical treatment in the Third Medical Center of PLA General Hospital from January 1996 to January 2018 were retrospectively analyzed. According to the presence or absence of nipple depression before surgery, the patients were divided into the nipple depression group and the non-nipple depression group. In the nipple depression group, patients were subdivided into a novel corrective surgery group (“one” suture or half pocket suture) and a conventional corrective surgery group (oil yarn traction valgus correction of nipple depression). Demographic, clinical, therapeutic, and postoperative relapse data were collected and analyzed.

**Results:**

Compared with the patients in the non-nipple depression group, patients in the nipple depression group had a significantly higher recurrence risk after surgery (HR = 2.129 95% CI: 1.110–4.083, *p* = 0.023). Patients who underwent novel corrective surgery had a significantly lower recurrence risk than those who underwent conventional corrective surgery (HR = 0.363 95% CI: 0.150–0.880, *p* = 0.025). In addition, the novel corrective surgery significantly reduced the postoperative recurrence risk (HR = 0.088 95% CI: 0.009–0.886, *p* = 0.037).

**Conclusion:**

How to correct nipple depression is a critical factor for postoperative recurrence of PCM, and this novel corrective surgery for nipple depression can effectively reduce the postoperative recurrence rate in patients with nipple depression.

## 1. Introduction

Plasma cell mastitis (PCM), also known as mammary duct ectasia or periductal mastitis (1), is a breast disease characterized by aseptic and chronic inflammatory manifestations. It primarily occurs in non-lactating women and is characterized by non-periodic breast pain, nipple discharge, nipple sag, and an areola mass. Microscopically, it is characterized by breast duct dilation and plasma cell infiltration (2, 3). The onset of acute breast lumps rapidly increase with redness and swelling, skin ulceration, and the flow of purulent secretions, and most do not heal for a long time. The pathogenesis of PCM is not clear at present. The reported pathogenesis factors include trauma, nipple sag, nipple discharge, abnormal development of mammary ducts, infection, receiving estrogen drugs, endocrine disorders, etc. (3).

Nipple depression is both a cause and a symptom of PCM. When PCM patients also have nipple depression, the shape and tissue structure of the breast can be damaged. Poor treatment will easily lead to breast rupture and fistula formation, and the disease course will be prolonged, will not heal, or can even worsen. Most of the patients with nipple depression are congenital nipple depression, and a few are caused by secondary inflammation, which occurs in unilateral or bilateral nipple deformity. The nipple is partially or completely buried in the areola, which is common in women (4). The treatment methods for PCM mainly include drug therapy and surgical therapy. For patients with depressed nipples, this is difficult to cure by drug therapy alone, and surgery is needed to correct the depressed nipples and treat PCM. The principle of surgical treatment of depressed nipples is to preserve the sensation and lactation function of patients as much as possible and maintain a good shape of postoperative nipples (4). However, the conventional surgical method is not ideal for the correction of nipple sag, the nipple may retract again, and relapse is common after surgery. To solve the problem of recurrence and obtain better nipple correction, we analyzed the data of surgical patients in our hospital over the past more than 20 years, enrolled 282 patients with PCM, and conducted a retrospective study of the patients with postoperative recurrence. Based on conventional surgery, we developed a new corrective method for nipple depression: one suture or half pocket suture. Based on our previous studies, we further analyzed postoperative cases to determine the therapeutic effect of the new corrective surgery on patients with PCM nipple depression.

## 2. Materials and methods

### 2.1. Study population

This study included PCM patients who underwent surgery at the third Medical Center of Chinese PLA General Hospital between January 1996 and June 2018. All patients were confirmed to have PCM by pathological diagnosis. Inclusion criteria were as follows: (1) Female patients; (2) The duration of the disease exceeded 1 month; and (3) Patients who received surgical treatment. Exclusion criteria were: (1) Male patients; (2) Patients with a tumor; and (3) Patients with underlying diseases such as liver and kidney dysfunction. According to the inclusion and exclusion criteria, a total of 282 patients were included and 55 patients were excluded.

### 2.2. Data collection

Clinical data were collected, and the age, treatment history, preoperative course of the disease, local symptoms and related risk factors in patients with PCM were recorded. The follow-up duration was from the date of PCM diagnosis to the date of the last review. When the patient developed breast pain, lumps, redness and other symptoms in the same part of the breast on the same side or in different quadrants, this was considered a recurrence. Patients were routinely followed up for more than 3 months.

### 2.3. Surgical methods

**Figure 1:**
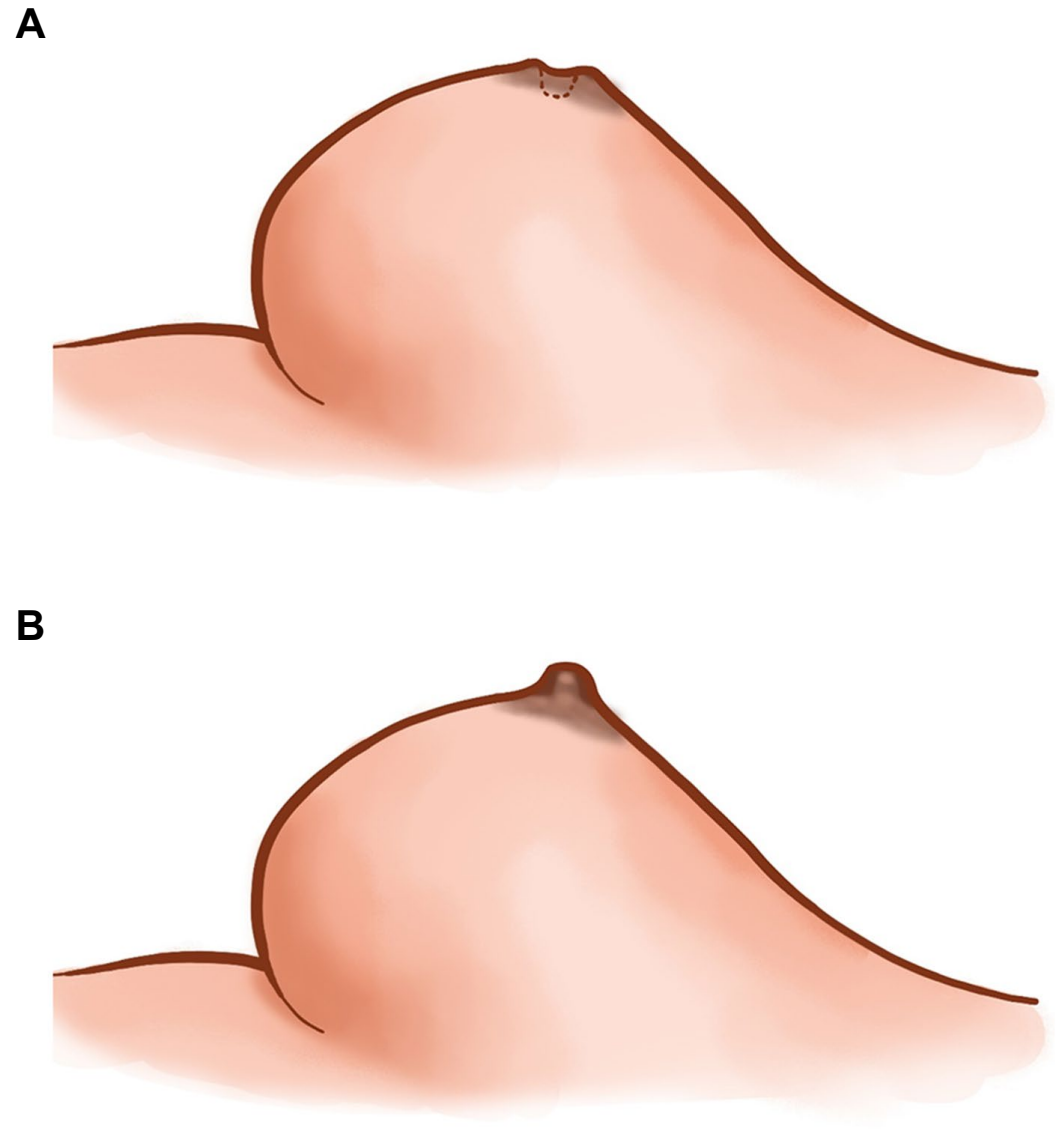
The plan of sunken nipple **(A)**, and plan of sunken eversion **(B)**.

Preoperatively, the surgical area in all patients was marked under color ultrasound and magnetic resonance imaging (MRI) guidance. The patient was placed on the operating table under general anesthesia, with both upper limbs abducted and the operative field fully exposed. Surgical incisions were made according to the preoperative positioning range. Most incisions were made along the areola, and radial incisions were made based on curved areola incisions with extensive lesions and difficulty in exposure to facilitate the resection of marginal lesions. If the skin was ruptured and the fistula needed to be removed together with the lesion, ruptured skin, and the fistula, removal of the fistula was sometimes guided by a probe. In patients with depressed nipples (Figure 1A), a valgus operation was performed on the depressed nipples at the same time as lesion resection.

**Figure 2:**
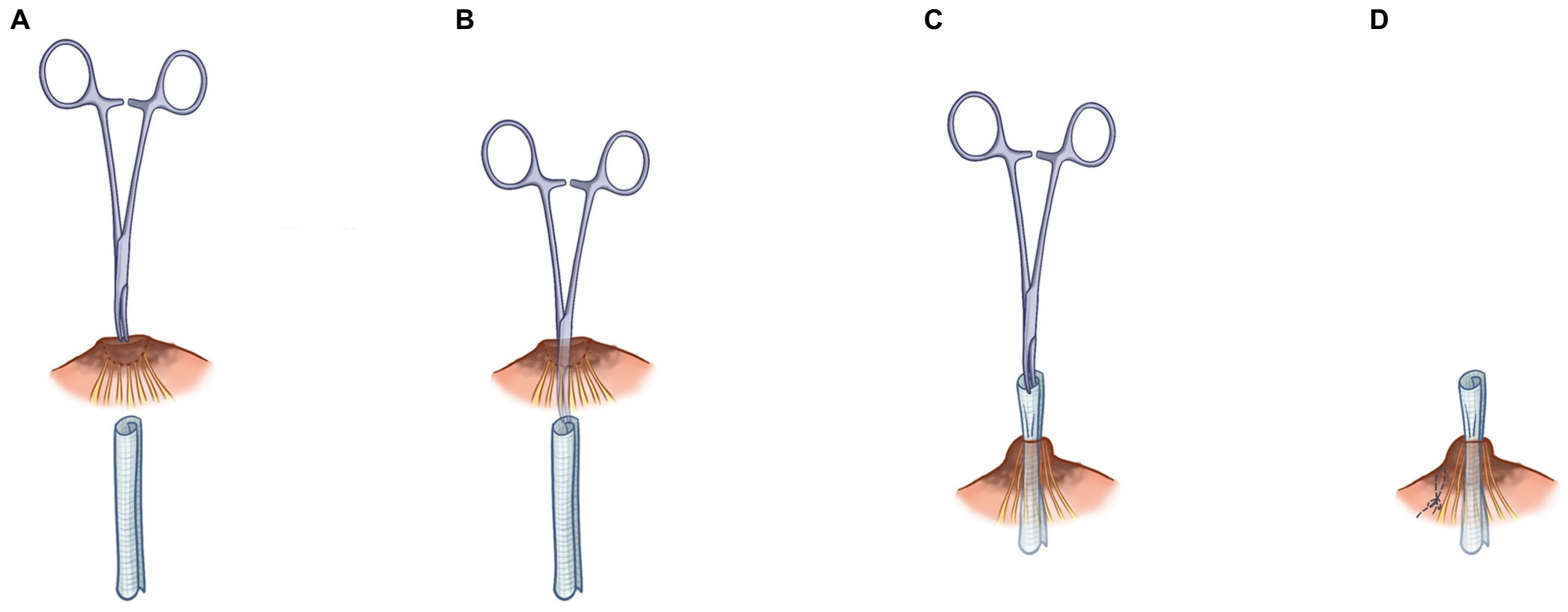
Step-by-step description of nipple depression valgus operation with traction. First place the mosquito forceps at the center of the sunken nipple **(A)**, then pull the mosquito forceps out of the center of the nipple **(B)**, then pull the oil yarn out of the center of the nipple **(C)**, and finally sew on the side of the nipple **(D)**.

Nipple depression valgus operation with traction was performed in the conventional corrective surgery (oil yarn traction valgus correction of nipple depression). During this procedure, the oil yarn strips were pulled out from the center of the nipple while the nipple was turned over, as shown in Figures 2A–D. The oil yarn strips were pulled out within 5 days after the operation. However, in some cases, the correction of the depressed nipple was unsuccessful, with postoperative recurrence; thus, the process requires improvement.

**Figure 3:**
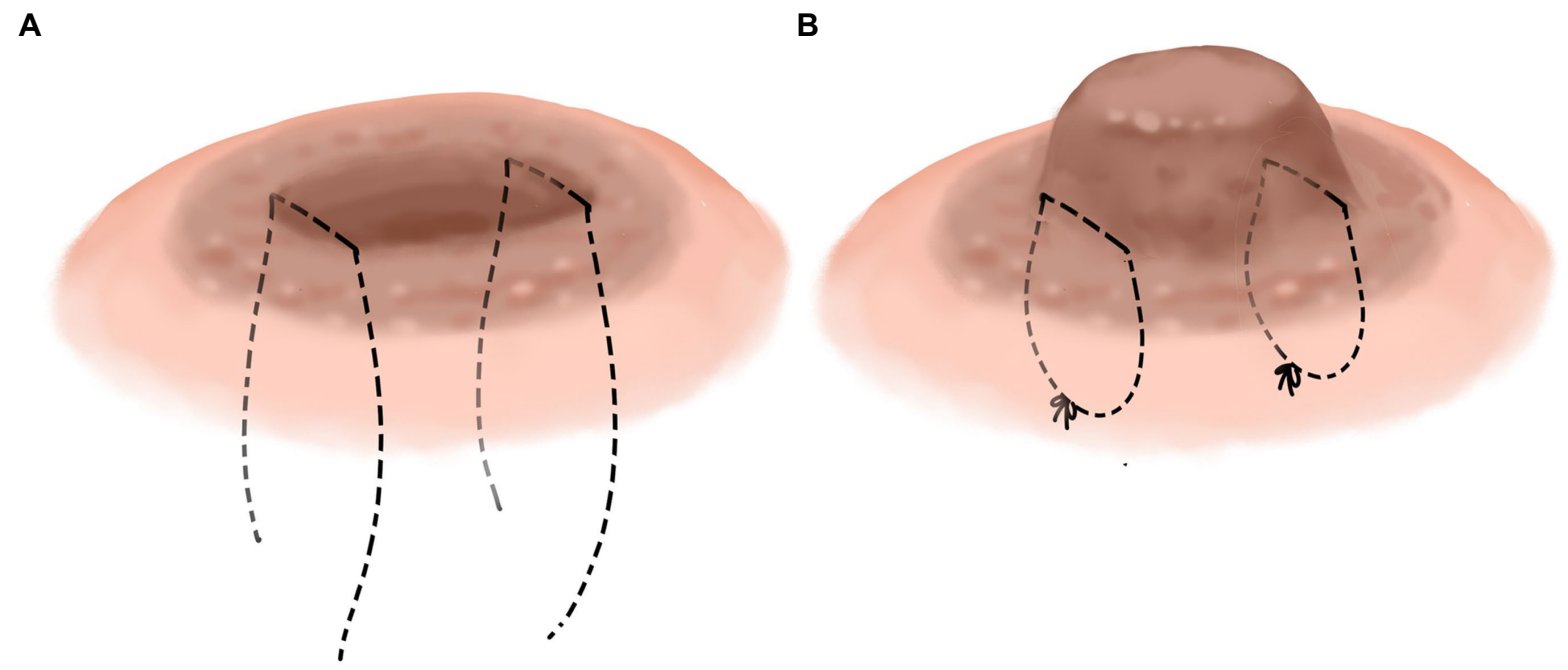
Nipple depression with perfect development. Sew “-” on both sides of the nipple root, respectively, **(A)** then turn the nipple out and tighten the knotted suture, respectively, **(B)**.

**Figure 4:**
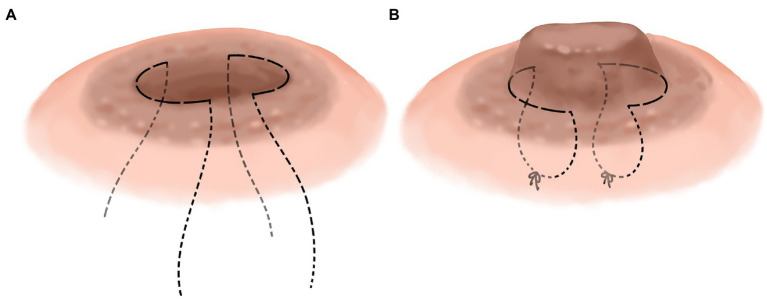
Nipple depression with imperfect development. Sew semicircular suture on both sides of the nipple root **(A)**, then gradually tighten the suture and tie the knot while turning the nipple outward **(B)**.

We improved the conventional corrective surgery. The novel corrective surgery is “-” suture or half pocket suture. Different surgical methods were used according to the different depression types. Nipple depression can be classified into two types according to its appearance: nipple depression with perfect development and nipple depression with imperfect development. The nipple with imperfect development was defined as part of the nipple being missing, or the nipple being too short. The well-developed novel surgical method involved everting the nipple and performing “-” suture on one side of the root of the nipple to evert the nipple (Figures 3A,B) completely. It is relatively difficult to complete eversion of the nipple due to the developmental defects of the nipple. The surgical method should be as far as possible to achieve the purpose of not depression the nipple, and the internal “half purse” suture and ligation should be performed. In the case of hyper- short and concave nipples, a partial fibrous bundle at the root of the nipple should be detached to lengthen the nipple and then sutured contralateral to complete nipple eversion (Figures 4A,B). Figure 1B shows the short-term effects after resection of the lesion and correction of the inverted nipple.

### 2.4. Pathology

**Figure 5:**
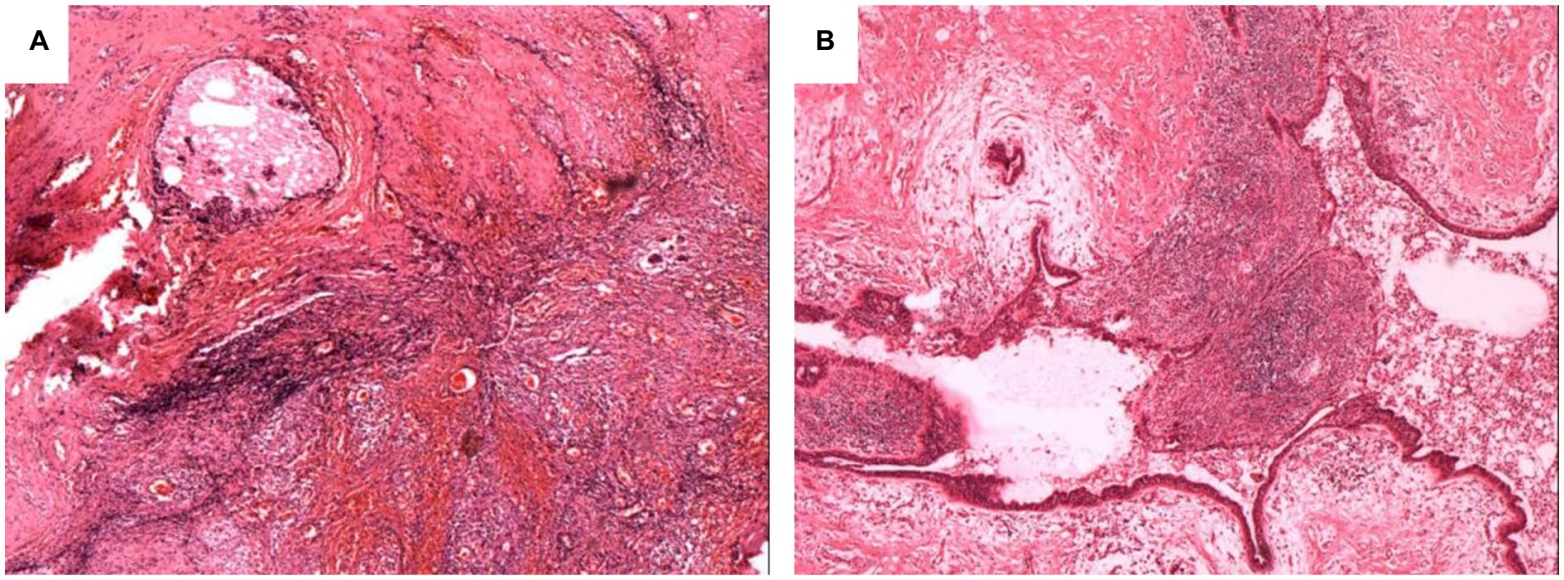
Pathological characteristics of PCM patient. More lymphocytes and plasma cells infiltrate around the large duct, and inflammatory cells can be seen in the lumen **(A,B)**.

The early pathological manifestations of this disease were irregular hyperplasia of ductal epithelium, ductal dilation, lumen enlargement, a large amount of epithelial cell debris and accumulation of lipid-containing secretions in the lumen, fibrosis of periductal tissues, and lymphocyte infiltration. In later-stage lesions, the wall of the catheter was thickened, fibrosis was observed, and small areas of focal fat necrosis were observed around the duct, with a large number of surrounding histocytes, neutrophils, lymphocytes, and plasma cells, especially plasma cells (Figures 5A,B).

### 2.5. Statistical analysis

SPSS 22.0 software was used to analyze the above data, and the significance level for all analyses was set at *p* < 0.05. The Kolmogorov–Smirnov test was used for normally distributed data. Categorical variables are expressed as frequency tables with percentages, and numerical variables conforming to normal distribution are expressed as mean ± standard deviation. When parametric conditions were provided, the independent sample *T*-test was used for inter-group comparisons. Chi-square analysis was used to test whether the categorical variables were correlated. Hazard ratios (HR) and 95% confidence intervals (CI) were calculated using the Logistic regression analysis. The variables included in multivariate analysis were significantly (*p* < 0.05) associated with recurrence in univariate analysis.

## 3. Results

A total of 282 patients with PCM who underwent surgical treatment were enrolled in this study. The onset age of the 282 patients was 16–61 years, the mean age was 33.17 ± 7.83 years, with an average BMI of 24.46 ± 4.68 kg/m^2^. Two cases (0.7%) had a history of smoking, and 23 cases (8.2%) had a history of breast trauma. The most common complaint was a breast mass (271 cases, 96.1%), 157 cases (55.7%) had breast redness and swelling, and 203 patients (72%) had history of breastfeeding. There were 140 patients (49.6%) with nipple depression, 41 patients (14.5%) with nipple discharge, and 12 patients (4.3%) with autoimmune diseases. A total of 134 patients (47.5%) underwent segmentectomy for breast lesions, and 138 (52.5%) underwent segmentectomy for breast lesions and correction of nipple depression. Fifty-two patients (18.4%) recorrected after surgery.

A total of 282 patients with PCM who underwent surgical treatment were included in this study, of whom 52 patients (18.4%) relapsed after surgical treatment. The data on the recurrent group and the non-recurrent group are shown in Table 1. History of smoking (*p* = 0.3353), breastfeeding (*p* = 0.7323), BMI (*p* = 0.2957), nipple discharge (*p* = 0.1335), redness and swelling (*p* = 0.8773), breast mass (*p* = 0.4315), breast trauma (*p* = 0.3969), and autoimmune diseases (*p* = 0.7019) were not significantly different between the two groups. More patients with younger age group (30.79 ± 6.76 vs. 33.76 ± 8.05, *p* = 0.0160), nipple depression (65.4% vs. 46.1%, *p* = 0.0139), ruptures (42.3% vs. 24.3%, *p* = 0.0154) and fistulas (9.6% vs. 1.3%, *p* = 0.0064) were observed in the recurrent group than in the non-recurrent group. Univariate and multivariate logistic regression analysis showed that nipple depression (HR = 2.129 95% CI: 1.110–4.083, *p* = 0.023) and fistulas (HR = 5.396 95% CI: 1.150–25.313, *p* = 0.033) were independent risk factors for recurrence (Table 2).

**Table 1:** Recurrence factors in PCM patients receiving surgical treatment.

	No-recurrent group (*N* = 230)	Recurrent group (*N* = 52)	Value of *p*
Age	33.76 ± 8.05	30.79 ± 6.76	**0.0160**
Smoking			0.3353
Yes	1 (0.4)	1 (1.9)	
No	229 (99.6)	51 (98.1)	
Breastfeeding			0.7326
Yes	66 (28.7)	13 (25.0)	
No	164 (71.3)	39 (75.0)	
BMI (kg/m^2^, mean ± SD)	24.45 ± 4.27	25.16 ± 4.60	0.2957
Nipple discharge			0.1335
Yes	37 (16.1)	4 (7.7)	
No	193 (83.9)	48 (92.3)	
Breast redness and swelling			0.8773
Yes	129 (56.1)	28 (53.8)	
No	101 (43.9)	24 (46.2)	
Breast mass			0.4315
Yes	222 (96.5)	49 (94.2)	
No	8 (3.5)	3 (5.8)	
Breast trauma			0.3969
Yes	17 (7.4)	6 (11.5)	
No	213 (92.6)	46 (88.5)	
Autoimmune diseases			0.7019
Yes	11 (4.8)	1 (1.9)	
No	219 (95.2)	51 (98.1)	
Rupture			**0.0154**
Yes	56 (24.3)	22 (42.3)	
No	174 (75.7)	30 (57.7)	
Fistulas			**0.0064**
Yes	3 (1.3)	5 (9.6)	
No	227 (98.7)	47 (90.4)	
Nipple depression			**0.0139**
Yes	106 (46.1)	34 (65.4)	
No	124 (53.9)	18 (34.6)	

Bolded *p* value indicates a statistical difference.

**Table 2:** Logistic regression for the predictors of recurrence in PCM patients receiving surgical treatment.

Variables	Univariate analyses		Multivariate analyses	
	HR (95% CI)	Value of *p*	HR (95% CI)	Value of *p*
Recurrence				
Age	1.056 (1.011–1.105)	**0.015**	1.043 (0.997–1.0921)	0.069
Smoking	4.490 (0.276–72.986)	0.291		
Breastfeeding	0.828 (0.416–1.651)	0.592		
BMI	0.958 (0.887–1.035)	0.273		
Nipple discharge	0.435 (0.148–1.279)	0.130		
Breast redness and swelling	0.913 (0.499–1.671)	0.769		
Breast mass	0.589 (0.151–2.299)	0.446		
Breast trauma	1.634 (0.611–4.371)	0.328		
Autoimmune diseases	0.390 (0.049–3.093)	0.373		
Rupture	2.279 (1.217–4.266)	**0.010**	1.749 (0.889–3.439)	0.105
Fistulas	8.050 (1.859–34.850)	**0.005**	5.396 (1.150–25.313)	**0.033**
Nipple depression	2.210 (1.180–4.138)	**0.013**	2.129 (1.110–4.083)	**0.023**

Bolded *p* value indicates a statistical difference.

We further compared the risk factors for postoperative recurrence in patients with inverted nipples. More ruptures (38.2% vs. 32.8%, *p* = 0.0497) and low novel surgery (26.5% vs. 46.2%, *p* = 0.0472) were observed in the recurrent group than in the non-recurrent group (Table 3). Univariate and multivariate logistic regression analysis showed that ruptures (HR = 2.803 95% CI: 1.207–6.509, *p* = 0.016) and surgery (HR = 0.363 95% CI: 0.150–0.880, *p* = 0.025) were independent risk factors for recurrence (Table 4).

**Table 3:** Factors of postoperative recurrence in PCM patients with nipple depression receiving surgical treatment.

	No-recurrent group (*N* = 106)	Recurrent group (*N* = 34)	Value of *p*
Age (years, mean ± SD)	33.10 ± 7.78	30.35 ± 7.11	0.0963
Smoking			0.2429
Yes	0 (0.0)	1 (2.9)	
No	106 (100.0)	33 (97.1)	
Breastfeeding			0.1864
Yes	33 (31.1)	6 (17.6)	
No	73 (68.9)	28 (82.4)	
BMI (kg/m^2^, mean ± SD)	25.05 ± 4.81	25.36 ± 4.84	0.7240
Nipple discharge			0.7819
Yes	16 (15.1)	4 (11.8)	
No	90 (84.9)	30 (88.2)	
Breast redness and swelling			0.4260
Yes	65 (61.3)	18 (52.9)	
No	41 (38.7)	16 (47.1)	
Breast mass			0.6327
Yes	102 (96.2)	32 (94.1)	
No	4 (3.8)	2 (5.9)	
Breast trauma			0.6878
Yes	8 (7.4)	1 (11.5)	
No	98 (92.6)	33 (88.5)	
Autoimmune diseases			>0.9999
Yes	3 (2.8)	1 (2.9)	
No	103 (97.2)	33 (97.1)	
Rupture			**0.0497**
Yes	26 (32.8)	15 (38.2)	
No	80 (67.2)	19 (61.8)	
Fistulas			0.2481
Yes	2 (1.9)	2 (5.9)	
No	104 (98.1)	32 (94.1)	
Surgery			**0.0472**
Novel	49 (46.2)	9 (26.5)	
Conventional	57 (53.8)	25 (73.5)	

Bolded *p* value indicates a statistical difference.

**Table 4:** Logistic regression for the predictors of recurrence in PCM patients with nipple depression receiving surgical treatment.

Variables	Univariate analyses		Multivariate analyses	
	HR (95% CI)	Value of *p*	HR (95% CI)	Value of *p*
Recurrence				
Age	1.054 (0.995–1.116)	0.073		
Smoking	NA	1.000		
Breastfeeding	0.474 (0.179–1.254)	0.133		
BMI	0.987 (0.894–1.089)	0.791		
Nipple discharge	0.750 (0.233–2.419)	0.630		
Breast redness and swelling	0.710 (0.326–1.546)	0.388		
Breast mass	0.627 (0.110–3.586)	0.600		
Breast trauma	0.371 (0.045–3.080)	0.359		
Autoimmune diseases	1.040 (0.105–10.345)	0.973		
Rupture	2.429 (1.082–5.454)	**0.031**	2.803 (1.207–6.509)	**0.016**
Fistulas	3.250 (0.440–24.005)	0.248		
Surgery	0.419 (0.179–0.982)	**0.045**	0.363 (0.150–0.880)	**0.025**

Bolded *p* value indicates a statistical difference.

Among the 34 patients with inverted nipple PCM patients who recurred after surgery, 26 patients received surgery again, and the other 8 patients received conservative treatment or returned to the local area for treatment. Therefore, we further evaluated the risk factors of postoperative recurrence in PCM patients after receiving secondary surgical treatment (Table 5). Compared with conventional corrective surgery, the novel surgery effectively reduced the postoperative recurrence rate (9.1% vs. 53.3%, *p* = 0.0362). Univariate logistic regression analysis showed that novel surgery (HR = 0.088 95% CI: 0.009–0.886, *p* = 0.037) were risk factors for postoperative recurrence in PCM patients after receiving secondary surgical treatment (Table 6).

**Table 5:** Factors of postoperative recurrence in PCM patients after receiving secondary surgical treatment.

	No-recurrent group (*N* = 17)	Recurrent group (*N* = 9)	Value of *p*
Age (years, mean ± SD)	31.24 ± 8.51	28.89 ± 6.75	0.8014
Smoking			>0.9999
Yes	0 (0.0)	0 (0.0)	
No	17 (100.0)	9 (100.0)	
Breastfeeding			>0.9999
Yes	3 (17.65)	1 (11.11)	
No	14 (82.35)	8 (88.89)	
BMI (kg/m^2^, mean ± SD)	25.27 ± 4.20	27.42 ± 7.24	0.6070
Nipple discharge			0.5292
Yes	2 (11.76)	0 (0.0)	
No	15 (88.24)	9 (100.0)	
Breast redness and swelling			0.4185
Yes	11 (64.7)	4 (44.4)	
No	6 (35.3)	5 (55.6)	
Breast mass			>0.9999
Yes	16 (94.12)	8 (88.89)	
No	1 (5.88)	1 (11.11)	
Breast trauma			>0.9999
Yes	16 (94.12)	8 (88.89)	
No	1 (5.88)	1 (11.11)	
Autoimmune diseases			>0.9999
Yes	1 (5.88)	0 (0.0)	
No	16 (94.12)	9 (100.0)	
Rupture			0.2167
Yes	9 (52.9)	2 (22.2)	
No	8 (47.1)	7 (77.8)	
Fistulas			0.5292
Yes	2 (11.76)	0 (0.0)	
No	15 (88.24)	9 (100.0)	
First surgery			0.1902
Novel	7 (41.2)	1 (11.1)	
Conventional	10 (58.8)	8 (88.9)	
Secondary surgery			**0.0362**
Novel	10 (58.8)	1 (11.1)	
Conventional	7 (41.2)	8 (88.9)	

Bolded *p* value indicates a statistical difference.

**Table 6:** Logistic regression for the predictors of recurrence in PCM patients after receiving secondary surgical treatment.

Variables	Univariate analyses	
	HR (95% CI)	Value of *p*
Recurrence		
Age	1.024 (0.919–1.141)	0.672
Smoking	NA	1.000
Breastfeeding	0.583 (0.052–6.587)	0.663
BMI	0.925 (0.756–1.132)	0.448
Nipple discharge	NA	0.999
Breast redness and swelling	0.436 (0.086–2.269)	0.324
Breast mass	0.500 (0.028–9.076)	0.639
Breast trauma	NA	1.000
Autoimmune diseases	NA	1.000
Rupture	0.254 (0.040–1.595)	0.144
Fistulas	NA	0.999
First surgery	0.179 (0.018–1.767)	0.141
Secondary surgery	0.088 (0.009–0.886)	**0.037**

Bolded *p* value indicates a statistical difference.

## 4. Discussion

PCM, also known as mammary duct ectasia, is a benign breast disease, accounting for ~4–5.3% of breast diseases, with an increasing trend year by year, mostly occurring in women under 40 years (5). At present, there is no consensus on the pathogenesis of PCM. Most scholars believe that PCM is an autoimmune disease caused by an abnormal autoimmune system. Other scholars believe that PCM is a secondary disease, and is usually secondary to bacterial infection, trauma, smoking, obesity and other high-risk factors (6, 7). Although studies have reported that the above factors can increase the risk of PCM, the exact cause is still unknown, and there is no precise treatment. Therefore, it is essential to identify the cause of PCM and provide targeted treatment.

This study showed that nipple depression may be a significant cause of postoperative recurrence in patients with PCM nipple depression. Palmieri A (8) reported a case of sunken nipples in her article on male plasmacytic mastitis. Most of the patients with open nipples were congenital, and a few were caused by secondary inflammation. Studies show that the direct cause of nipple depression is dysplasia of the smooth muscle of the nipple and areola caused by dysplasia of the nipple mesoderm in the early stage of nipple development, the loss of supporting tissue below the nipple and a reduction in fibrous connective tissue of the mammary duct and its surrounding area. As a result, the nipple is too short, the tissue does not support the nipple and ultimately leads to nipple depression (9). Nipple depression can directly affect the patency of the breast duct, leading to expansion of the breast duct (10, 11). In patients with nipple depression, the epidermal cells and secretions of the nipple cannot be discharged outside the body surface, resulting in the long-term accumulation of lipid-like secretions and their decomposition products in the duct in the moist and closed environment of the nipple depression, forming mixed mucus retrograde into the mammary duct. The long-term accumulation leads to blockage of the mammary duct, and to a certain extent, will overflow, and retrograde into the mammary duct. When the body resistance is decreased, disordered hormone levels in the body cause stimulation of phospholipid production inside the breast duct, inducing proliferation of duct wall tissue, a large amount of floccus attaches to the wall of the breast duct causing blockage of the breast duct, resulting in inflammation of the wall and surrounding tissue. This causes the mixed mucus in the breast duct to infiltrate the interstitium through the space between damaged epithelial cells, leading to inflammatory reactions and a series of symptoms such as breast redness, swelling or lumps. The production of many inflammatory substances clogging the breast duct forms a vicious cycle; Long-term blockage of the breast duct can cause bacterial colonization. When the external environment changes and the pressure in the milk duct further increases, this can induce a suppurative inflammatory reaction, leading to the occurrence of PCM (12–14). In this study, 140 of 282 patients had nipple depression, and 34 of 52 recurrent patients (65.4%) had nipple depression, which is a risk factor for the onset of PCM. Therefore, correcting nipple depression is crucial for patients with PCM.

The treatment of PCM has always been controversial. The main methods include drug and surgical therapy, of which surgical therapy is still the most thorough and effective treatment for PCM (15, 16). The principle of surgical treatment is to resect the lesion as much as possible. However, the high recurrence rate after surgery makes many surgeons shy away, as the surgical incision is long, the scope of resection is enormous, and the breast deformity caused by multiple operations is difficult for patients to accept. The key to surgical treatment is to remove the diseased ducts below the nipple and the involved glands (2) to reduce the chance of recurrence. However, there can be more than one blocked mammary duct. There are 16–18 mammary ducts in the human mammary gland. The mucus in the breast duct can continue to clog and aggravate the condition, coupled with the inevitable recurrence of the precipitating factors. Therefore, the probability of recurrence is high. The recurrence rate of PCM can be reduced by surgical resection of the lesion and correction of the depressed nipple. It can be seen from Table 1 that patients with inverted nipples have a higher recurrence rate, so whether the recurrence rate of PCM is related to the success of nipple eversion is debatable.

Nipple depression is generally classified into two types according to its appearance: nipple depression with perfect development and nipple depression with imperfect development. The nipple with imperfect development is defined as part of the nipple being missing or the nipple being too short. Nipple depression occurs in short milk ducts due to poor growth of the milk ducts and insufficient proliferation of interstitial lobes in the depression. To date, there is no consensus on the preferred treatment, which is divided into two categories: breast duct preservation and breast duct destruction. The most successful way to correct nipple depression is not to damage the milk duct and to preserve lactation function, with good cosmetic appearance of the nipple without retraction. Non-surgical treatment methods, such as rubber bands or external suction device fixation retain the milk ducts (17, 18). Surgical methods mainly include a true flap, traction, and minimally invasive and endoscopic release of fibrous tissue (19, 20).

However, as PCM is an inflammatory reaction, the correction of nipple depression cannot fully meet the above requirements. The focus of the operation is the success of valgus and the prevention of recurrence. In this study we started to correct the inverted nipple using oil yarn to pull the inverted nipple through the central hole, and inversion of the nipple was mostly successful. The main advantage of this method is that traction of the nipple eversion does not damage the milk duct, and the root of the nipple is not sutured. Due to the imperceptibility of the posterior nipple suture, multiple artificial large milk ducts are over-squeezed and narrow. If there is a large amount of storage in the expanded ducts, the pressure will rise sharply, aggravating the duct injury and leading to recurrence. In this study, the oil gauze protected the nipple and stimulated the growth of interstitial tissue behind the nipple. With the increase in the number of surgical and recurrent cases, it was found that this surgical method has defects, and a few patients with imperfect nipple development still had recurrence after surgery. With novel corrective surgery, as nipple depression is mostly due to the nipple duct being too short, the surgical method in the root of the nipple lateral line an up and down or left and right suture can avoid nipple retraction, to achieve complete eversion of the nipple. This method also protects the function of the milk duct, avoids damage to the milk duct, and avoids the discomfort caused by removal of the oil yarn after the operation. For the underdeveloped nipple due to developmental defects, partial loss of the nipple, lack of capacity of the missing part caused by depression, a complete eversion of the nipple is relatively tricky, thus as far as possible, to achieve nipple eversion, internal “half purse” suture ligation should be performed. In the case of excessively short and concave nipples caused by the partial absence of both nipples and milk ducts, partial fiber bundles at the root of the nipple should be separated to extend the nipple and then sutured to the lateral edge of the opposite side to achieve nipple eversion. Before this procedure, storage in the central milk duct should be emptied as far as possible.

This study also has some limitations. First, this is a retrospective study, which might have selection biases. Second, this was a single-center study and the number of included cases was limited. Nevertheless, our study showed that after the novel corrective surgery, the success rate of nipple eversion was higher, and the recurrence risk was lower than that of the conventional corrective surgery. Future multicenter studies of the effectiveness of this novel corrective surgery should be conducted in order to maximize patient benefit.

## 5. Conclusion

The purpose of this study was to review the treatment of patients and analyze the therapeutic effect of the novel corrective surgery for depressed nipple in patients with PCM. The recurrence rate following this novel nipple corrective surgery was 15% lower than that of the conventional corrective surgery. This novel nipple corrective surgery effectively reduced the postoperative recurrence rate in patients with an inverted depression.

## Data availability statement

The original contributions presented in the study are included in the article/supplementary material, further inquiries can be directed to the corresponding author.

## Ethics statement

The studies involving human participants were reviewed and approved by Institute Research Ethics Committees of the 3rd Medical Center of Chinese PLA General Hospital. The patients/participants provided their written informed consent to participate in this study. Written informed consent was obtained from the individual(s) for the publication of any potentially identifiable images or data included in this article.

## Author contributions

HX and BD came up with the concept and designed the experiments. YW, FZ, and LX collected the cohort data and samples. HS, MG, and LH analyzed the data. HX, XL, and BD contributed reagents/materials/analysis tools. YX, HZ, and SM completed the manuscript. HX made extensive revisions to the manuscript. All authors contributed to the article and approved the submitted version.

## Conflict of interest

The authors declare that the research was conducted in the absence of any commercial or financial relationships that could be construed as a potential conflict of interest.

## Publisher’s note

All claims expressed in this article are solely those of the authors and do not necessarily represent those of their affiliated organizations, or those of the publisher, the editors and the reviewers. Any product that may be evaluated in this article, or claim that may be made by its manufacturer, is not guaranteed or endorsed by the publisher.
